# Clinically Silent, Metastatic Renal Cell Carcinoma Detected on Routine Screening Mammogram: A Report of a Rare Case and Review of Literature

**DOI:** 10.7759/cureus.48352

**Published:** 2023-11-06

**Authors:** Elizabeth Pernicone, Kelly Fabrega-Foster

**Affiliations:** 1 Radiology, Saint Francis Medical Center, Peoria, USA; 2 Breast Imaging, Tampa General Hospital, Tampa, USA

**Keywords:** breast metastasis, extra-mammary malignancy, renal cell carcinoma, oncology, breast imaging

## Abstract

Renal cell carcinoma (RCC) is the most common and the most lethal urogenital malignancy, and can metastasize rapidly via hematogenous spread. Even so, RCC metastasis within the breast is extremely rare and may appear deceptively benign on screening mammograms. In this article, we present a rare case of RCC that remained undiagnosed until an intra-mammary metastasis was detected on a routine screening mammogram. Further imaging workup and core needle biopsy of the mass ultimately confirmed a new diagnosis of metastatic clear cell RCC. Given that the presence of an RCC breast metastasis indicated advanced-stage RCC, the patient in this case underwent treatment with systemic immunotherapy. This case report describes key imaging features of metastatic RCC on common breast imaging modalities. It underscores the vital role that screening mammography can play in the initial detection of clinically silent, extra-mammary malignancies, including RCC. Thorough imaging workup and tissue biopsy are essential to distinguish a primary breast lesion from intra-mammary metastatic disease, to inform the management plan, and to prevent lumpectomy or mastectomy when it does not benefit the patient.

## Introduction

Renal cell carcinoma (RCC) is the seventh most common malignancy in the Western world, and is both the most common and the most lethal urogenital malignancy [1]. RCC is an insidious neoplasm that can spread to any organ system via hematogenous spread, but metastasis to the breast is extremely uncommon, and breast imaging findings in the setting of metastatic RCC can be somewhat non-specific, necessitating tissue biopsy for definitive diagnosis. It is important to distinguish metastatic RCC from a primary breast malignancy, as the treatment plan and prognosis differ widely for these two conditions [2]. In this case report and literature review, we describe an unusual case of clinically silent metastatic RCC that was detected incidentally as a new breast mass on routine screening mammogram.

## Case presentation

**Figure 1:**
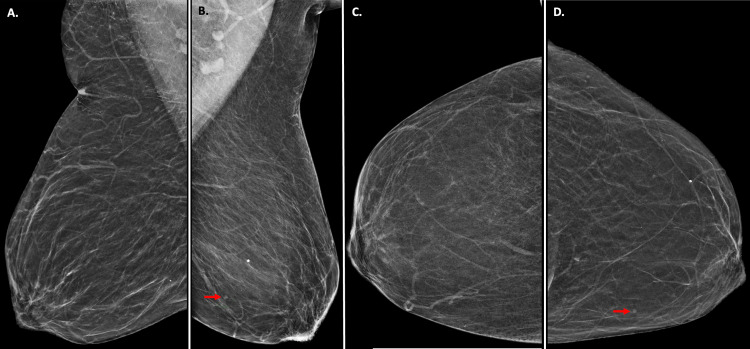
3D Screening Mammogram Findings (A) Mediolateral oblique view and (C) craniocaudal view show unremarkable mammographic findings in the patient’s right breast. (B) Mediolateral oblique view and (D) craniocaudal view show a small, circumscribed mass (red arrows) in the medial left breast. This mass was not seen on previous mammograms.

A 76-year-old female patient presented to the breast imaging center for her annual screening mammogram. Her past medical history included hypothyroidism, bilateral simple renal cysts, stage III chronic kidney disease, and chronic hypertension. She had no history of prior tobacco use. Her family history included pancreatic cancer in her maternal grandmother but was otherwise negative for malignancy. At the time of her presentation for screening mammogram, she voiced no complaints; however, she was quite overdue for mammographic screening, having not undergone an annual mammogram for three years. The screening mammogram demonstrated fatty breasts, with a small, 2.5 mm mass observed in the medial left breast at eight o’clock, that had not appeared on any of her previous mammograms (Figure 1). Although the mass was relatively innocuous-appearing, with circumscribed borders and no associated distortion or calcifications, its recent development prompted the radiologist to call back the patient for additional imaging.

**Figure 2:**
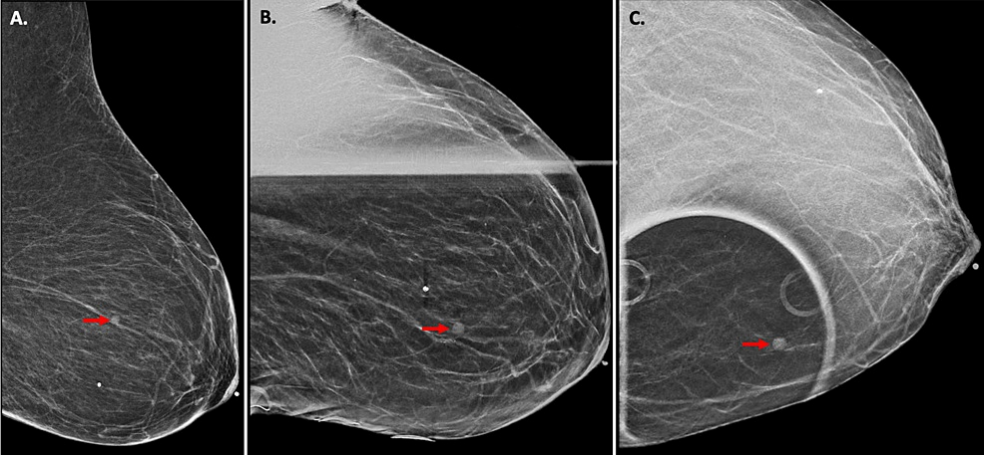
Left Diagnostic Mammogram (A) Mediolateral view of the left breast; the mass (red arrows) that had been previously visualized on a screening mammogram one month prior to this exam had apparently doubled in size. The mass did not efface with compression, as demonstrated in: (B) mediolateral oblique compression view and (C) craniocaudal compression view.

**Figure 3:**
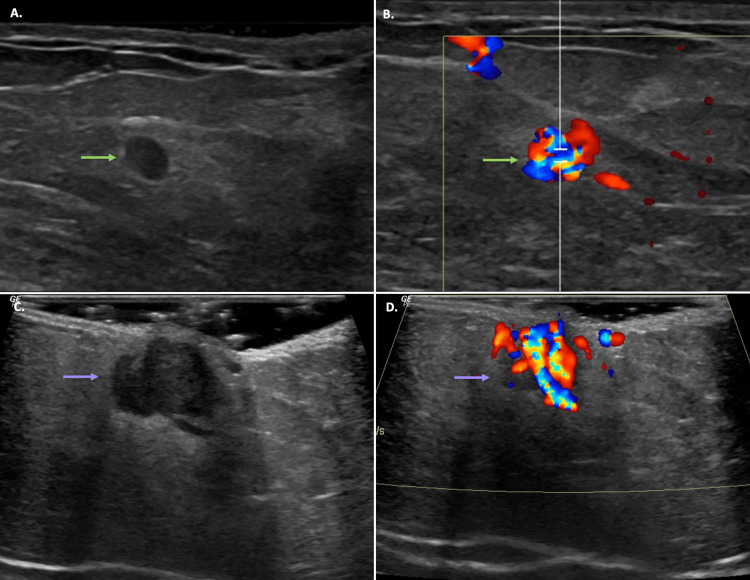
Left Breast and Axillary Ultrasound Findings (A, B) Hypoechoic, circumscribed, round, hypervascular mass (lime green arrows) is seen in the left breast at 10:00, 3-4 cm from the nipple, which corresponded to the mass seen on mammogram. (C, D) A second, larger, more superficial mass (lavender arrows) is seen in the left axilla. The left axillary mass was hypoechoic and hypervascular, similar in appearance to the 10 o' clock left breast mass.

The patient returned one month later, at which time a diagnostic mammogram and targeted left breast ultrasound were performed to further evaluate the left breast focal asymmetry. On the diagnostic mammogram, it was noted that the previously visualized medial left breast mass had doubled in size, and now measured 5 mm in greatest dimension (Figure 2). Its borders still appeared to be well-circumscribed, and there was still no associated distortion and no calcifications within or surrounding the mass. On ultrasound, in the left breast at the ten o’clock position, the lesion appeared as a circumscribed, hypoechoic, round, 5 mm mass with significant internal vascularity (Figure 3). Ultrasound-guided biopsy was recommended.

Three weeks later, the patient returned to the breast imaging center for an ultrasound-guided left breast biopsy. During her conversation with the radiologist prior to the biopsy, the patient mentioned that for approximately one to two years prior to this time, she had noticed multiple small nodules developing under her skin in multiple areas throughout her body, and had recently noticed a new palpable lump in her left axilla. The radiologist physically examined the area and noted a palpable, superficial, mobile nodule, which was subtly bluish in gross appearance. Ultrasound examination of this left axillary mass revealed a superficial, extremely vascular, non-parallel, fairly circumscribed mass with posterior acoustic shadowing (Figure 3). It somewhat resembled an inflamed sebaceous cyst; however, no tract to the epidermis was visible. Surgical excision was recommended.

**Figure 4:**
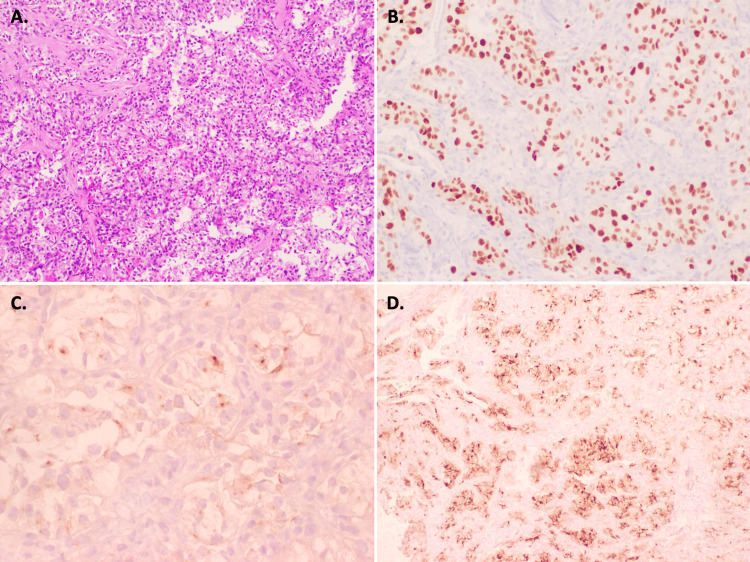
Left Breast Mass Core Biopsy Histology (A) Nests of neoplastic cells with clear cytoplasm with interspersed blood vessels, hematoxylin and eosin staining; Immunohistochemical staining of the tissue demonstrated positive staining for PAX8 (B), RCC (C), and CD10 (D). This is consistent with a diagnosis of metastatic clear cell carcinoma, from a renal origin. RCC: renal cell carcinoma

She underwent the ultrasound-guided biopsy of her medial left breast mass, which notably bled rather significantly during the biopsy and, despite appropriate compression of the left breast after the biopsy, developed a small post-biopsy hematoma. The final pathology of the left breast mass biopsy demonstrated clear cell carcinoma compatible with metastatic clear cell renal carcinoma (Figure 4).

**Figure 5:**
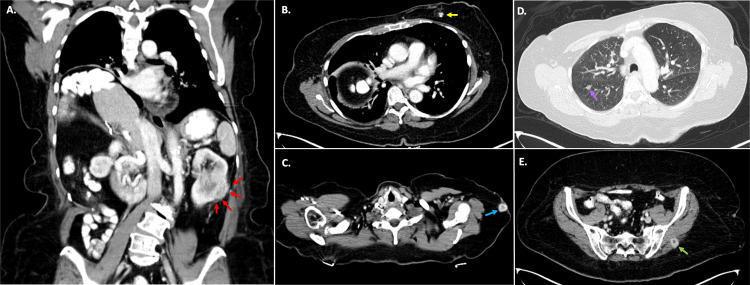
Contrast-Enhanced CT of the Chest, Abdomen, and Pelvis (A) Renal cell carcinoma primary lesion (red arrows), a large, heterogeneously enhancing mass invading the lower pole of the left kidney but which did not appear to involve the inferior vena cava, the left renal vein, or the left renal collecting system. (B, C) Left breast metastatic lesion (yellow arrow), which was first observed on screening mammogram, and the left axillary subcutaneous metastasis (blue arrow), which was discovered on ultrasound exam when the patient presented for biopsy of her breast mass. (D, E) Additional circumscribed, enhancing metastatic lesions in the lungs (lavender arrow) and in the gluteus muscle (green arrow).

The patient was promptly referred for medical and surgical oncology management. She underwent contrast-enhanced computed tomography (CT) scanning of the chest, abdomen, and pelvis for further staging. This revealed a large, heterogeneously enhancing lesion involving the lower pole of the left kidney. No left renal vein invasion, no retroperitoneal lymphadenopathy, and no involvement of the left renal pelvis was observed. A metastatic lesion was visualized involving the left breast, in addition to numerous additional lesions within the lungs, subcutaneous tissues, and even skeletal muscle (Figure 5). Given the advanced stage of her disease, the patient was not a candidate for surgical resection. She ultimately pursued systemic therapy with pembrolizumab and lenvatinib, with complete resolution of her metastatic lesions and a 50% reduction in the size of her primary malignancy reported on the six-month follow-up. 

## Discussion

RCC is the most common urogenital malignancy and is also the most lethal, with a 76% five-year relative survival rate. The survivability of this disease depends largely on the disease stage at the time of diagnosis; the five-year survival drops from 93% in the setting of stage 1, localized disease down to 12% in patients with stage IV metastatic disease. Men are more commonly affected than women; two-thirds of all cases are diagnosed in men. RCC is most typically found in older patients, with an average age of 64 years at the time of diagnosis, although it may occur at younger ages in patients with hereditary renal cell cancer syndromes such as von Hippel-Lindau syndrome or tuberous sclerosis. Other, modifiable risk factors for RCC include tobacco use, obesity, chronic hypertension, and certain occupational exposures such as trichloroethylene [1].

Clinically, RCC may present in a classic symptom triad of hematuria, flank pain, and palpable renal masses [3]. However, this “textbook” presentation occurs in only 4-17% of patients who are diagnosed [3,4]. In fact, RCC often remains clinically silent for years, leading to delays in diagnosis and treatment; it is most commonly first detected as an incidental finding on abdominal imaging. Of newly diagnosed patients, 30% present with metastatic disease, most commonly in the lungs, skeleton, or liver [5]. Metastasis to the breast is extremely rare and has only been reported in a few prior case reports, and in all but one of those case reports, the patients had a known history of RCC and were presenting with recurrent, metastatic disease [6-10]. Our patient had no known history of RCC prior to her breast biopsy and other than the palpable nodules that she reported at the time of her breast biopsy, she was asymptomatic at the time of diagnosis.

Imaging findings on mammography and ultrasound can be somewhat non-specific, sharing certain features with other types of intra-mammary metastases and with benign breast masses [11-14]. On mammography, metastatic RCC typically presents as a circumscribed, round or oval-shaped mass that may appear deceptively benign, due to a lack of spiculations or internal microcalcifications. Often, intra-mammary metastases do not involve the breast ducts and therefore may not cause significant distortion on imaging. On ultrasound, RCC in the breast usually appears as a hypoechoic, circumscribed, round or oval-shaped mass, with marked internal vascularity and no posterior acoustic shadowing. Previous studies have also described the appearance of metastatic RCC within the breast on other imaging modalities such as CT, 18F-fluorodeoxyglucose (FDG) positron emission tomography (PET)/CT, and magnetic resonance imaging (MRI) [9-17]. On CT, it is typically a dense, circumscribed, enhancing mass within the breast, without spiculations and without nipple retraction or skin involvement. Because breast metastasis from RCC signifies stage IV disease, other metastatic lesions may be visible on chest or abdominal CT. PET/CT findings may show mild-moderately increased uptake of FDG in RCC metastases within the breast and other tissues, although its utility is limited in the evaluation of primary RCC lesions [10,15,16]. MRI may show a circumscribed, round or oval breast mass that appears hypo-intense on T1 sequence, hyperintense on fat-saturated T2 sequence, and that is rapidly enhancing with plateau kinetics upon administration of gadolinium contrast [9,17].

Definitive diagnosis of RCC is accomplished with tissue biopsy. Histologic examination differentiates several RCC subtypes, including clear cell, type 1 and 2 papillary, chromophobe, collecting duct, or medullary [18-20]. Clear cell RCC, the subtype found in our patient’s case, is the most common subtype, and histologic exam of this subtype typically demonstrates nests or sheets of cells with prominent, lipid-rich cytoplasm and abundant, interspersed blood vessels. Immunohistochemical staining is essential to confirm the diagnosis of RCC and to accurately categorize the many different RCC subtypes [19,21]. In our patient’s case, increased PAX-8 expression in the tumor tissue indicated a renal origin, increased RCC expression confirmed the diagnosis of RCC, and positive CD10 expression hinted at a clear cell histologic subtype.

The prompt and accurate diagnosis of RCC metastasis in the breast is vital for deciding on a suitable treatment plan; while resection of a primary breast mass with lumpectomy or mastectomy is appropriate, surgical resection of an RCC metastasis in the breast may result in unnecessary surgical morbidity without providing a meaningful therapeutic benefit [22,2]. Appropriate management of RCC depends on the stage at the time of diagnosis. While localized RCC can be effectively cured with partial or radical nephrectomy, metastatic RCC is better managed with systemic therapy, including targeted therapy with anti-angiogenics and immunotherapy [23,24]. Radiation therapy may be useful for patients with localized disease when complete surgical resection is not possible, or as a palliative therapy in patients with widely metastatic disease [25].

## Conclusions

RCC is an insidious neoplasm that can spread to every organ system via hematogenous spread, but metastasis to the breast is extremely uncommon, and breast imaging findings in the setting of metastatic RCC can be somewhat non-specific, necessitating tissue biopsy for definitive diagnosis. In this case report, we presented and discussed key imaging findings that are typically observed in metastatic RCC lesions within the breast; these imaging features are often similar amongst metastatic breast lesions from many different extramammary sources. The confirmation of RCC metastasis within the breast heralds advanced disease and indicates the need for systemic therapy, including targeted therapy with anti-angiogenics and/or immunotherapy. Prompt imaging workup and tissue biopsy are essential to distinguish a primary breast malignancy from intra-mammary metastasis, to inform the management plan, and to prevent lumpectomy or mastectomy when it does not benefit the patient. 
